# A Glutamine Insertion at Codon 432 of RpoB Confers Rifampicin Resistance in *Mycobacterium tuberculosis*

**DOI:** 10.3389/fmicb.2020.583194

**Published:** 2020-10-19

**Authors:** Li-Yin Lai, Li-Yu Hsu, Shang-Hui Weng, Shuo-En Chung, Hui-En Ke, Tzu-Lung Lin, Pei-Fang Hsieh, Wei-Ting Lee, Hsing-Yuan Tsai, Wan-Hsuan Lin, Ruwen Jou, Jin-Town Wang

**Affiliations:** ^1^Department of Microbiology, National Taiwan University College of Medicine, Taipei, Taiwan; ^2^Tuberculosis Research Center, Centers for Disease Control, Ministry of Health and Welfare of Taiwan, Taipei, Taiwan; ^3^Center for Diagnostics and Vaccine Development, Centers for Disease Control, Ministry of Health and Welfare of Taiwan, Taipei, Taiwan; ^4^Department of Internal Medicine, National Taiwan University Hospital, Taipei, Taiwan

**Keywords:** *Mycobacterium tuberculosis*, drug resistance, rifampicin, *rpoB*, codon 432 of RpoB

## Abstract

Tuberculosis (TB) is an infectious respiratory disease caused by *Mycobacterium tuberculosis* and one of the top 10 causes of death worldwide. Treating TB is challenging; successful treatment requires a long course of multiple antibiotics. Rifampicin (RIF) is a first-line drug for treating TB, and the development of RIF-resistant *M. tuberculosis* makes treatment even more difficult. To determine the mechanism of RIF resistance in these strains, we searched for novel mutations by sequencing. Four isolates, CDC-1, CDC-2, CDC-3, and CDC-4, had high-level RIF resistance and unique mutations encoding RpoB G^158^R, RpoB V^168^A, RpoB S^188^P, and RpoB Q^432^insQ, respectively. To evaluate their correlation with RIF resistance, plasmids carrying *rpoB* genes encoding these mutant proteins were transfected into the H_37_Rv reference strain. The plasmid complementation of RpoB indicated that G^158^R, V^168^A, and S^188^P did not affect the MIC of RIF. However, the MIC of RIF was increased in H_37_Rv carrying RpoB Q^432^insQ. To confirm the correlation between RIF resistance and Q^432^insQ, we cloned an *rpoB* fragment carrying the insertion (encoding RpoB Q^432^insQ) into H_37_Rv by homologous recombination using a suicide vector. All replacement mutants expressing RpoB Q^432^insQ were resistant to RIF (MIC > 1 mg/L). These results indicate that RpoB Q^432^insQ causes RIF resistance in *M. tuberculosis*.

## Introduction

*Mycobacterium tuberculosis* is an important human pathogen that causes tuberculosis (TB). It was first isolated by Robert Koch in 1882, and numerous TB drugs have been developed since the 1930s, although the first effective anti-TB drug was not discovered until 1944 (Vilcheze and Jacobs, 2014; Islam et al., 2017; Long et al., 2019; Mabhula and Singh, 2019). TB is a common, chronic infectious respiratory disease that affects nearly one-third of the world’s population (Long et al., 2019; Yong et al., 2019) and is transmitted via aerosol (e.g., cough) from infected persons. According to the World Health Organization, TB is one of the top 10 causes of death worldwide. In 2018, there were approximately 10 million people with TB and 1.4 million deaths due to TB worldwide (World Health Organization [WHO], 2019). Approximately 500,000 new cases were found to be resistant to the most effective first-line drug, rifampicin (RIF), of which 78% had multidrug-resistant TB (resistant to at least isoniazid and RIF) (World Health Organization [WHO], 2019). According to the Taiwan Centers for Disease Control, there were 9,179 new cases of TB in Taiwan in 2018, and 294 of these were RIF-resistant. Over the period of long-term anti-TB therapy, *M. tuberculosis* is exposed to the appropriate drug concentration, which might lead to the development of drug-resistant TB and increase the risk of transmission (Yong et al., 2019). It takes at least 6 months to successfully treat TB, and the development of drug resistance makes therapy even more difficult and is a threat to public health. The treatment of drug-resistant TB requires the administration of more than five drugs for more than 9 months (Vekemans et al., 2020). In addition, RIF-resistant TB is frequently not adequately treated because of a delay in the diagnosis of drug resistance. Such delayed treatment not only has a poor therapeutic effect on the infected patient but also increases the risk of transmission (Boyd et al., 2017; Yong et al., 2019). First-line RIF acts by binding the β-subunit of RNA polymerase, blocking RNA synthesis, and inducing hydroxyl radical formation, which likely contributes to its killing effect (Piccaro et al., 2014; Lohrasbi et al., 2018). It has been reported that drug resistance develops when mutations in *rpoB* block RIF binding to the β-subunit. Most point mutations causing RIF resistance occur in an 81-nucleotide region (codons 426–452 in *M. tuberculosis* or codons 507–533 in the *Escherichia coli* codon numbering system) of *rpoB* that is called the RIF resistance-determining region (RRDR) (Pang et al., 2013; Chikaonda et al., 2017). According to previous studies, >90% of RIF-resistant TB strains have mutations in the RRDR within codon 435, 445, and 450 (codon 516, 526, and 531 in *E. coli*) (Pang et al., 2013; Swain et al., 2020). Drug resistance is a major challenge for TB control. Due to the slow growth rate of *M. tuberculosis*, conventional drug susceptibility testing takes several weeks. Therefore, sequencing drug resistance-related mutations can be used to quickly detect drug-resistant TB, significantly reducing the duration of therapy, and avoiding treatment delays. Thus, we examined the effects of novel mutations in known target gene regions and mutations outside of the target region on RIF resistance in *M. tuberculosis* with the aim to expand upon known RIF resistance-causing mutations for use in clinical molecular diagnostics.

## Materials and Methods

### Bacteria Strains

Clinical isolates were obtained from the Reference Laboratory of Mycobacteriology of the Taiwan Centers for Disease Control. *M. tuberculosis* H_37_Rv was used as the reference strain. *M. tuberculosis* H_37_Rv and clinical isolates were cultured in Difco^TM^ Middlebrook 7H9 medium (BD REF: 271310; MD, NJ, United States) supplemented with 10% oleic acid/albumin/dextrose/catalase (OADC), 0.5% glycerol, and 0.05% tween-80 at 37°C (Tan et al., 2006). *E. coli* DH10B was grown in Luria broth (LBL405.1; BioShop, Burlington, Canada). All experiments involving *M. tuberculosis* strains were conducted at the Biosafety level 3 lab in the National Taiwan University College of Medicine and followed institutional biosafety procedures.

### Isolation of Clinical Strains and Drug Susceptibility Testing

Clinical isolates of *M. tuberculosis* were tested in the Reference Laboratory of Mycobacteriology of the Taiwan Centers for Disease Control. Each isolate was initially obtained from a patient and was inoculated in solid and liquid culture. The minimum inhibitory concentration (MIC) of RIF was determined using a Sensititre^TM^ Mycobacterium tuberculosis MIC Plate (MYCOTB; Thermo Fisher Scientific, Cleveland, OH, United States) according to the manufacturer’s instructions. The bacterial culture was adjusted to a 0.5 McFarland standard and then added to the Sensititre^TM^ plate, which was covered with an adhesive plastic seal. After incubation at 37°C, the results were recorded with a Sensititre^TM^ Vizion^TM^ Digital MIC Viewing System. The critical concentration of RIF was 1 mg/L.

### Microplate Alamar Blue Assays (MABAs)

Microplate alamar blue assays were performed as previously described with minor modifications (Burke et al., 2017). Briefly, *M. tuberculosis* strains were cultured in Middlebrook 7H9 medium, and then the OD_600__nm_ was adjusted to 0.1. A 200-μL aliquot of each prepared bacterial suspension was placed in a 96-well sterile plate (LabServ, Singapore) and incubated at 37°C for 14 days with and without RIF (0.03–512 mg/L). After 14 days of incubation, 50 μL of a freshly prepared 1:1 mixture of Alamar blue (alamarBlue^®^; BioRad, Hercules, CA, United States) and 10% tween 80 was added to each well. Then, the plate was incubated at 37°C for 48 h, and the color was recorded. Blue indicates no growth, and pink indicates growth. During incubation and staining, the plate was sealed with an adhesive plate seal.

### RpoB Expression Plasmids

The plasmid pMN437 was used to express RpoB in *M. tuberculosis* (Steinhauer et al., 2010). Derivative plasmids carrying resistance-associated *rpoB* mutations were generated using the QuikChange II Site-Directed Mutagenesis Kit (Agilent, Santa Clara, CA, United States). The primers used are shown in Supplementary Table 1. *M. tuberculosis* was transformed by electroporation at 2,500 V, 1,000 Ω, and 25 μF and recovered in 10 mL of Middlebrook 7H9 medium supplemented with 10% OADC and 0.5% glycerol for 24 h (Larsen et al., 2007; Lai et al., 2018). Recovered cells were then plated on BBL^TM^ 7H11 solid agar (BD REF: 212203; BD, MD, NJ, United States) containing 50 mg/L hygromycin and incubated at 37°C for 4 weeks.

### Site-Directed Mutagenesis

The putative resistance-associated *rpoB* mutations were amplified by PCR from the genomic DNA of the resistant strains as a template and then cloned into the *Sca*I site of the suicide plasmid pGOAL19 (Addgene; MA, United States) (Parish and Stoker, 2000). The primers used are shown in Supplementary Table 1. The resulting plasmids were transformed into *M. tuberculosis* H_37_Rv, and point mutants were selected after two rounds of homologous recombination, as previously described (Parish and Stoker, 2000; Larsen et al., 2007).

### DNA Sequencing

*Mycobacterium tuberculosis* isolates were lysed with InstantGet^TM^ DNA Extraction Solution (Catalog Number: 17001; HNG, Taipei, Taiwan) and heat-killed (100°C for 15 min). Then, the supernatants of heat-killed *M. tuberculosis* isolates were added to the PCR reagent containing KOD Xtreme^TM^ Hot Start DNA Polymerase (Catalog Number: 71976; Novagen, CA, United States) and gene-specific primers, rpoB out F1 and rpoB-out-R (Supplementary Table 1), to amplify the *rpoB* fragment. The PCR products were sent for sequencing (Mission Biotech, Taipei, Taiwan) and were verified by capillary electrophoresis (ABI 3730xl; Thermo Fisher Scientific, Cleveland, OH, United States). The *rpoB* sequences of clinical strains CDC-1, CDC-2, and CDC-4 were submitted to NCBI (GenBank accession numbers: MT774526, MT774527, and MT774528, respectively).

## Results

### Identification of Novel *rpoB* Mutations in RIF-Resistant Clinical Isolates

Four clinical isolates, CDC-1, CDC-2, CDC-3, and CDC-4, had high-level RIF resistance and different novel mutations within the *rpoB* gene. The RIF resistance levels of clinical strains were determined using a Sensititre^TM^ Mycobacterium tuberculosis MIC Plate. The MIC of RIF for CDC-1 was 8 mg/L, and the MICs of RIF for CDC-2, CDC-3, and CDC-4 were >16 mg/L (Table 1). Sequencing showed that each of these four clinical isolates harbored genes encoding two amino acid changes in RpoB. When compared to the sequence of the reference strain H_37_Rv, in CDC-1, the G at codon 158 was replaced with R (G^158^R) and the V at codon 170 was replaced with F (V^170^F). In CDC-2, the V at codon 168 was replaced with A (V^168^A) and the V at codon 170 was replaced with F (V^170^F). In CDC-3, the V at codon 170 of RpoB was substituted with F (V^170^F), and the S at codon 188 was replaced with P (S^188^P). In CDC-4, the S at codon 431 was replaced with G (S^431^G) and a Q was inserted at codon 432 (Q^432^insQ). However, G^158^R, V^168^A, S^188^P, and Q^432^insQ had not previously been confirmed to be related to drug resistance.

**TABLE 1:** Rifampicin (RIF) resistance levels of clinical strains as measured by Sensititre^TM^ Mycobacterium tuberculosis MIC Plate.

**Strains**	**RpoB substitutions**	**RpoB Codon change**	**MIC of RIF (mg/L)**
CDC-1	G^158^R & V^170^F	158 GGC → CGC	8
		170 GTC → TTC	
CDC-2	V^168^A & V^170^F	168 GTG → GCG	>16
		170 GTC → TTC	
CDC-3	V^170^F & S^188^P	170 GTC → TTC	>16
		188 TCC → CCC	
CDC-4	S^431^G & Q^432^insQ	431 AGC → GGC	>16
		432 CAA → CAACAA	

*RIF resistance: >1mg/L. MIC, minimum inhibitory concentration.*

### An RpoB Q^432^insQ Expression Plasmid Increases the MIC of RIF in H_37_Rv

**FIGURE 1:**
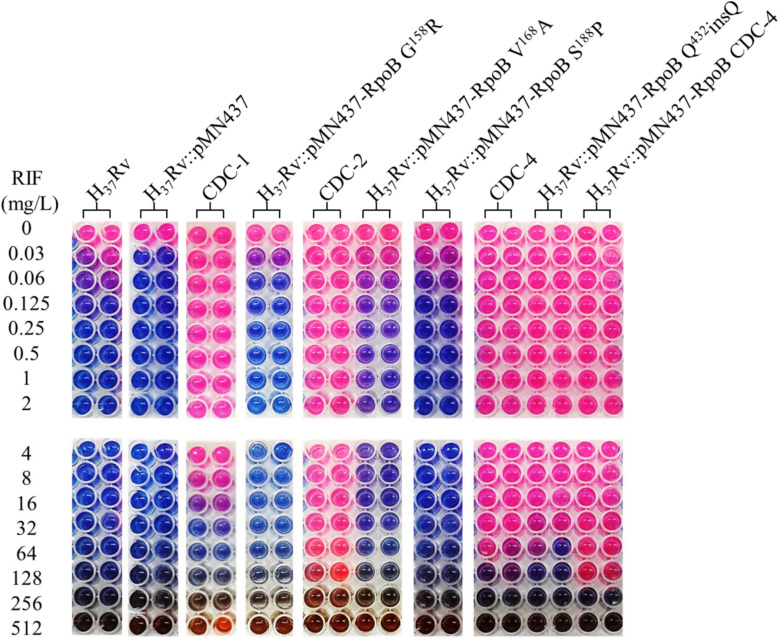
Rifampicin (RIF) susceptibility test for *Mycobacterium tuberculosis* strains expressing wild-type and mutant RpoB as measured by microplate Alamar blue assays (MABAs). The MIC of RIF for *M. tuberculosi*s strains carrying RpoB expression plasmids was measured by MABA. *M. tuberculosis* was cultured in 7H9 broth containing different concentrations of RIF for 2 weeks and then stained with Alamar blue for 2 days. CDC-1, CDC-2, and CDC-4 are RIF-resistant clinical strains that harbored RpoB amino acid changes (G^158^R & V^170^F, V^168^A & V^170^F, and S^431^G & Q^432^insQ, respectively). Red indicates bacterial growth, and blue indicates no growth. The critical concentration for RIF resistance was1.0 mg/L. All experiments were performed in duplicate to confirm reproducibility and repeated at least twice with similar results (Table 3).

Clinical isolates CDC-1, CDC-2, CDC-3, and CDC-4 had high-level RIF resistance and harbored unique RpoB amino acid changes (G^158^R, V^168^A, S^188^P, and Q^432^insQ, respectively). To evaluate the association between these unique amino acid changes and the RIF resistance of the clinical isolates, plasmids (pMN437-derived) expressing these RpoB proteins were transformed into H_37_Rv (Table 2), and RIF susceptibility was measured in these strains by a MABA. The results showed that the MICs of RIF for the wild-type strains, H_37_Rv and H_37_Rv:pMN437, were 0.03–0.125 mg/L. The MIC of RIF for clinical strain CDC-1 was 4–32 mg/L, that for clinical strain CDC-2 was 256–512 mg/L, and that for clinical strain CDC-4 was 128–256 mg/L. These results were consistent with the MIC data obtained with the Sensititre^TM^ Mycobacterium tuberculosis MIC Plate. However, the MIC of RIF for clinical strain CDC-3 could not be determined by the MABA because it could not be cultured. The MIC of RIF for H_37_Rv:pMN437-RpoB G^158^R, H_37_Rv:pMN437-RpoB V^168^A, and H_37_Rv:pMN437-RpoB S^188^P was 0.03–0.25 mg/L, that for H_37_Rv:pMN437-CDC-4 was 128–256 mg/L, and that for H_37_Rv:pMN437-RpoB Q^432^insQ was 64–128 mg/L (Tables 2, 3, Figure 1). These results showed that H_37_Rv:pMN437-RpoB G^158^R, H_37_Rv:pMN437-RpoB V^168^A, and H_37_Rv:pMN437-RpoB S^188^P were not resistant to RIF (MIC < 1 mg/L). The MIC of RIF for H_37_Rv:pMN437-CDC-4 was the same as that of clinical strain CDC-4, and the MIC of RIF for H_37_Rv:pMN437-RpoB Q^432^insQ was two-fold lower than that for H_37_Rv:pMN437-CDC-4 and clinical strain CDC-4. This suggests that RpoB G^158^R, RpoB V^168^A, and RpoB S^188^P likely do not contribute to RIF resistance, whereas RpoB Q^432^insQ contributes to high-level RIF resistance in *M. tuberculosis*.

**TABLE 2:** Plasmids used in this study.

**Plasmids**	Amino acid substitutions*	**Target strain**	**Predicted consequence**	**Obtained phenotypic consequences**
pMN437-RpoB G^158^R	RpoB G^158^R	H_37_Rv	S → R	S → S
pMN437-RpoB V^168^A	RpoB V^168^A	H_37_Rv	S → R	S → S
pMN437-RpoB S^188^P	RpoB S^188^P	H_37_Rv	S → R	S → S
pMN437-RpoB-CDC-4	RpoB S^431^G & Q^432^insQ	H_37_Rv	S → R	S → R
pMN437-RpoB Q^432^insQ	RpoB Q^432^insQ	H_37_Rv	S → R	S → R
pGOAL19-Rv RpoB Q^432^insQ	RpoB Q^432^insQ	H_37_Rv	S → R	S → R

S, sensitive strain; R, resistant strain. * Rifampicin resistance-related amino acid substitutions.

**TABLE 3:** Susceptibility test results of clinical isolates and H_37_Rv strains carrying RpoB expression plasmids as measured by microplate Alamar blue assays.

**Strains**	**RpoB substitutions**	**MIC of RIF (mg/L)**		
		**Exp. 1**	**Exp. 2**	**Exp. 3**
H_37_Rv	None	0.125	0.03	0.125
H_37_Rv:pMN437	None	0.03	0.03	0.125
CDC-1	V^170^F & G^158^R	32	4	–
H_37_Rv:pMN437-RpoB G^158^R	G^158^R*	0.06	0.125	–
CDC-2	V^170^F & V^168^A	256	512	–
H_37_Rv:pMN437-RpoB V^168^A	V^168^A*	0.125	0.25	–
H_37_Rv:pMN437-RpoB S^188^P	S^188^P*	0.06	0.03	–
CDC-4	S^431^G & Q^432^insQ	128	128	256
H_37_Rv:pMN437-RpoB Q^432^insQ	Q^432^insQ*	128	64	128
H_37_Rv:pMN437-RpoB-CDC-4	S^431^G & Q^432^insQ*	256	128	256

Exp., experiment; MIC, minimum inhibitory concentration. * Amino acid substitutions in RpoB based on pMN437. ^–^Was not included in the experiment. RIF resistance: >1 mg/L.

### RpoB Q^432^insQ Confers RIF Resistance to *M. tuberculosis* H_37_Rv

**FIGURE 2:**
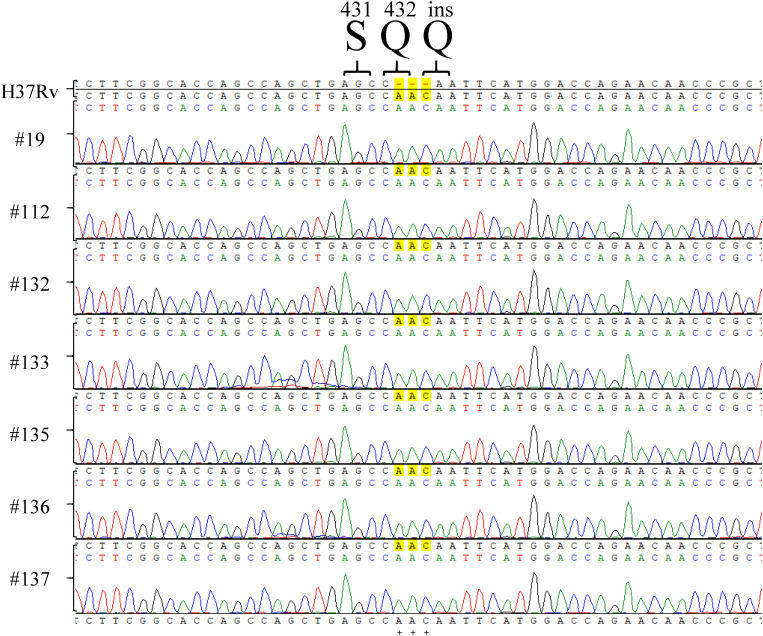
*rpoB* sequencing results of *Mycobacterium tuberculosis* H_37_Rv RpoB Q^432^insQ strains generated by site-directed mutagenesis. Sequencing of *rpoB* gene fragments of the strains generated by site-directed mutagenesis, specifically #9, #112, #132, #133, #135, #136, and #137, showed that RpoB was successfully substituted with RpoB Q^432^insQ in *M. tuberculosis* H_37_Rv-derived strains. H_37_Rv: *M. tuberculosis* H_37_Rv. Green pick: adenine, A. Red pick: thymine, T. Blue pick: cytosine, C. Black pick: guanine, G.

Among the tested RpoB expression plasmids, only RpoB Q^432^insQ increased the MIC of RIF in H_37_Rv. To further confirm that the RpoB Q^432^insQ was correlated with RIF resistance, unmarked mutants of H_37_Rv expressing RpoB Q^432^insQ were generated. The *rpoB* fragment with the CAA insertion was subcloned from pMN437-RpoB Q^432^insQ into the *Sca*I site of the pGOAL19 plasmid, and the resulting construct, pGOAL19-Rv RpoB Q^432^insQ (Table 2) was checked by sequencing. Then, the plasmid was transformed into *M. tuberculosis* H_37_Rv, and the unmarked replacement mutant (RpoB Q^432^insQ) was selected after two rounds of homologous recombination. DNA sequencing confirmed that seven strains (#19, #112, #132, #133, #135, #136, and #137) had the CAA insertion in codon 432 of *rpoB*, which leads to a Q insertion (Figure 2). All tested strains were resistant to RIF, with an MIC >1 mg/L.

## Discussion

Clinical strains CDC-1, CDC-2, and CDC-3 have high-level RIF resistance and unique *rpoB* gene mutations. All of them also encoded V^170^F, which is a well-known RIF-resistance associated mutation, as it has been reported that *M. tuberculosis* isolates encoding the V^170^F mutation have high-level resistance to RIF (8–32 mg/L) (Heep et al., 2000). According to our data, G^158^R, V^168^A, and S^188^P mutations likely did not contribute to RIF resistance in these strains. However, strain CDC-2 had a RIF MIC of 256–512 mg/L, which was 32–64 fold higher than the MIC previously reported for a strain encoding RpoB V^170^F (Heep et al., 2000), suggesting that strain CDC-2 might have another drug-resistance mechanism not involving RpoB. Because we only sequenced the *rpoB* genes of RIF-resistant strains, resistance mechanisms outside *rpoB* could not be detected. Mutations causing other antibiotic resistance could be missed too. Chromosome mutations comprise the major mechanism causing drug resistance in *M. tuberculosis* (Miotto et al., 2018). Drug resistance in strains could be caused by other resistance mechanisms such as antibiotic modifications or neutralization, augmented efflux pumps, porin alterations, and the downregulation of cell-wall permeability (Mnyambwa et al., 2017; Sharma et al., 2018).

Codons 431 and 432 of *rpoB* are located in the RRDR (Friehs, 2004), and mutations in this region are related to RIF resistance (Sandgren et al., 2009). Mutations at codon 431 (S^431^T, S^431^I, S^431^R, and S^431^G) were previously reported to be associated with RIF resistance (Kapur et al., 1994; Sajduda et al., 2004; Chan et al., 2007). Multiple mutations including codon 431 or 432 of RpoB resulted in higher-level RIF resistance (MIC > 100 mg/L) (Bahrmand et al., 2009). We also demonstrated that plasmid complementation or chromosomal mutagenesis of Q^432^insQ alone could cause RIF resistance, whereas S^431^G plus Q^432^insQ complementation resulted in a two-fold higher MIC of RIF (Tables 2, 3, Figure 1).

RIF interacts with the β-subunit of RpoB, and most RIF resistance in *M. tuberculosis* is caused by mutations in the RRDR of RpoB (Tupin et al., 2010). The domain structure of RpoB was previously deduced by analyzing the crystal structure, which showed that RpoB directly interacts with RIF via 12 hydrogen bonds (Nusrath Unissa et al., 2016). Molecular docking experiments showed stronger RIF binding by wild-type RpoB than by mutant RpoB proteins (Nusrath Unissa et al., 2016). Q432 is an energetically favorable binding site and is considered part of the active site that is involved in ligand binding (Campbell et al., 2001; Nusrath Unissa et al., 2016). RpoB Q^432^insQ might influence the binding enthalpy to weaken the molecular interaction between RpoB and RIF, which could result in RIF resistance.

We observed that CDC-4 grew slower compared to the H_37_Rv strain. Therefore, mutants survived better only in the presence of RIF. Multiple mutations in *rpoB* have been reported, shown to result in increased drug resistance (Bahrmand et al., 2009). The occurrence of multiple mutations could be accumulated due to continuous RIF usage after a first mutation or they could occur simultaneously in high RIF environments.

Rifampicin is an effective drug used to treat most cases of drug-susceptible TB (Long et al., 2019). However, cases of RIF resistance have been reported since the early 1990s, leading to problems in TB control (Zaw et al., 2018). In *M. tuberculosis*, drug resistance is mostly caused by genetic changes rather than gene transfer from other bacteria (Tupin et al., 2010; Zaw et al., 2018). Thus, sequencing well-known mutation sites is important to detect drug-resistant *M. tuberculosis*. Several molecular diagnostic methods have been developed for the rapid detection of drug resistance in *M. tuberculosis* (Yong et al., 2019). Nevertheless, more comprehensive information on drug resistance-associated mutations must be established to improve the diagnosis and treatment of TB.

## Conclusion

In summary, we studied four isolates with high-level RIF resistance and unique mutations encoding RpoB G^158^R, RpoB V^168^A, RpoB S^188^P, and RpoB Q^432^insQ. Results of plasmid complementation of RpoB indicated that G^158^R, V^168^A, and S^188^P of RpoB do not affect the MIC of RIF. However, the transfer of pMN437-RpoB Q^432^insQ plasmids to *M. tuberculosis* H_37_Rv or chromosomal mutagenesis generating RpoB Q^432^insQ turned sensitive strains into RIF-resistant strains. Therefore, RpoB Q^432^insQ confers RIF resistance in *M. tuberculosis*.

## Data Availability Statement

The datasets presented in this study can be found in online repositories. The names of the repository/repositories and accession number(s) can be found in the article/Supplementary Material.

## Author Contributions

J-TW designed the study. L-YL, P-FH, T-LL, and J-TW discussed the results and revised the manuscript. W-TL, H-YT, W-HL, and RJ provided and analyzed the clinical strains. L-YL, S-HW, S-EC, L-YH, and H-EK prepared materials and performed experiments. L-YL and P-FH analyzed the data. L-YL wrote the manuscript. All authors reviewed and approved the final version of the manuscript.

## Conflict of Interest

The authors declare that the research was conducted in the absence of any commercial or financial relationships that could be construed as a potential conflict of interest.

## References

[B1] Bahrmand A. R. Titov L. P. Tasbiti A. H. Yari S. Graviss E. A. (2009). High-level rifampin resistance correlates with multiple mutations in the rpoB gene of pulmonary tuberculosis isolates from the Afghanistan border of Iran. *J. Clin. Microbiol.* 47 2744–2750. 10.1128/jcm.r00548-09 19721079 PMC2738065

[B2] Boyd R. Ford N. Padgen P. Cox H. (2017). Time to treatment for rifampicin-resistant tuberculosis: systematic review and meta-analysis. *Int. J. Tuberc. Lung Dis.* 21 1173–1180. 10.5588/ijtld.17.0230 29037299 PMC5644740

[B3] Burke R. M. Coronel J. Moore D. (2017). Minimum inhibitory concentration distributions for first- and second-line antimicrobials against *Mycobacterium tuberculosis*. *J. Med. Microbiol.* 66 1023–1026. 10.1099/jmm.0.000534 28759352

[B4] Campbell E. A. Korzheva N. Mustaev A. Murakami K. Nair S. Goldfarb A. (2001). Structural mechanism for rifampicin inhibition of bacterial rna polymerase. *Cell* 104 901–912. 10.1016/s0092-8674(01)00286-0 11290327

[B5] Chan R. C. Hui M. Chan E. W. Au T. K. Chin M. L. Yip C. K. (2007). Genetic and phenotypic characterization of drug-resistant *Mycobacterium tuberculosis* isolates in Hong Kong. *J. Antimicrob. Chemother.* 59 866–873. 10.1093/jac/dkm054 17360809 PMC5404905

[B6] Chikaonda T. Ketseoglou I. Nguluwe N. Krysiak R. Thengolose I. Nyakwawa F. (2017). Molecular characterisation of rifampicin-resistant *Mycobacterium tuberculosis* strains from Malawi. *Afr. J. Lab. Med.* 6:463. 10.4102/ajlm.v6i2.463 PMC5523914 28879159

[B7] Friehs K. (2004). Plasmid copy number and plasmid stability. *Adv. Biochem. Eng. Biotechnol.* 86 47–82. 15088763 10.1007/b12440

[B8] Heep M. Rieger U. Beck D. Lehn N. (2000). Mutations in the beginning of the rpoB gene can induce resistance to rifamycins in both *Helicobacter pylori* and *Mycobacterium tuberculosis*. *Antimicrob. Agents Chemother.* 44 1075–1077. 10.1128/aac.44.4.1075-1077.2000 10722516 PMC89817

[B9] Islam M. M. Hameed H. M. A. Mugweru J. Chhotaray C. Wang C. Tan Y. (2017). Drug resistance mechanisms and novel drug targets for tuberculosis therapy. *J. Genet. Genom.* 44 21–37. 10.1016/j.jgg.2016.10.002 28117224

[B10] Kapur V. Li L. L. Iordanescu S. Hamrick M. R. Wanger A. Kreiswirth B. N. (1994). Characterization by automated DNA sequencing of mutations in the gene (rpoB) encoding the RNA polymerase beta subunit in rifampin-resistant *Mycobacterium tuberculosis* strains from New York City and Texas. *J. Clin. Microbiol.* 32 1095–1098. 10.1128/jcm.32.4.1095-1098.1994 8027320 PMC267194

[B11] Lai L. Y. Lin T. L. Chen Y. Y. Hsieh P. F. Wang J. T. (2018). Role of the *Mycobacterium marinum* ESX-1 secretion system in sliding motility and biofilm formation. *Front. Microbiol.* 9:1160. 10.3389/fmicb.2018.01160 29899738 PMC5988883

[B12] Larsen M. H. Biermann K. Tandberg S. Hsu T. Jacobs W. R. Jr. (2007). Genetic manipulation of *Mycobacterium tuberculosis*. *Curr. Protoc. Microbiol. Chap.* 10:Unit 10A 12. 10.1002/9780471729259.mc10a02s6 18770603

[B13] Lohrasbi V. Talebi M. Bialvaei A. Z. Fattorini L. Drancourt M. Heidary M. (2018). Trends in the discovery of new drugs for *Mycobacterium tuberculosis* therapy with a glance at resistance. *Tuberculosis* 109 17–27. 10.1016/j.tube.2017.12.002 29559117

[B14] Long B. Liang S. Y. Koyfman A. Gottlieb M. (2019). Tuberculosis: a focused review for the emergency medicine clinician. *Am. J. Emerg. Med.* 38 1014–1022. 10.1016/j.ajem.2019.12.040 31902701

[B15] Mabhula A. Singh V. (2019). Drug-resistance in *Mycobacterium tuberculosis*: where we stand. *Med. Chem. Commun.* 10 1342–1360. 10.1039/c9md00057g 31534654 PMC6748343

[B16] Miotto P. Zhang Y. Cirillo D. M. Yam W. C. (2018). Drug resistance mechanisms and drug susceptibility testing for tuberculosis. *Respirology* 23 1098–1113. 10.1111/resp.13393 30189463

[B17] Mnyambwa N. P. Kim D. J. Ngadaya E. S. Kazwala R. Petrucka P. Mfinanga S. G. (2017). Clinical implication of novel drug resistance-conferring mutations in resistant tuberculosis. *Eur. J. Clin. Microbiol. Infect. Dis.* 36 2021–2028. 10.1007/s10096-017-3027-3 28593375

[B18] Nusrath Unissa A. Hassan S. Indira Kumari V. Revathy R. Hanna L. E. (2016). Insights into RpoB clinical mutants in mediating rifampicin resistance in *Mycobacterium tuberculosis*. *J. Mol. Graph. Model.* 67 20–32. 10.1016/j.jmgm.2016.04.005 27155814

[B19] Pang Y. Lu J. Wang Y. Song Y. Wang S. Zhao Y. (2013). Study of the rifampin monoresistance mechanism in *Mycobacterium tuberculosis*. *Antimicrob. Agents Chemother.* 57 893–900. 10.1128/aac.01024-12 23208715 PMC3553728

[B20] Parish T. Stoker N. G. (2000). Use of a flexible cassette method to generate a double unmarked *Mycobacterium tuberculosis* tlyA plcABC mutant by gene replacement. *Microbiology* 146(Pt 8), 1969–1975. 10.1099/00221287-146-8-1969 10931901

[B21] Piccaro G. Pietraforte D. Giannoni F. Mustazzolu A. Fattorini L. (2014). Rifampin induces hydroxyl radical formation in *Mycobacterium tuberculosis*. *Antimicrob. Agents Chemother.* 58 7527–7533. 10.1128/aac.03169-14 25288092 PMC4249506

[B22] Sajduda A. Brzostek A. Poplawska M. Augustynowicz-Kopec E. Zwolska Z. Niemann S. (2004). Molecular characterization of rifampin- and isoniazid-resistant *Mycobacterium tuberculosis* strains isolated in Poland. *J. Clin. Microbiol.* 42 2425–2431. 10.1128/jcm.42.6.2425-2431.2004 15184414 PMC427864

[B23] Sandgren A. Strong M. Muthukrishnan P. Weiner B. K. Church G. M. Murray M. B. (2009). Tuberculosis drug resistance mutation database. *PLoS Med.* 6:e1000002. 10.1371/journal.pmed.1000002 19209951 PMC2637921

[B24] Sharma D. Bisht D. Khan A. U. (2018). Potential alternative strategy against drug resistant tuberculosis: a proteomics prospect. *Proteomes* 6:26. 10.3390/proteomes6020026 29843395 PMC6027512

[B25] Steinhauer K. Eschenbacher I. Radischat N. Detsch C. Niederweis M. Goroncy-Bermes P. (2010). Rapid evaluation of the mycobactericidal efficacy of disinfectants in the quantitative carrier test EN 14563 by using fluorescent *Mycobacterium terrae*. *Appl. Environ. Microbiol.* 76 546–554. 10.1128/aem.01660-09 19948860 PMC2805218

[B26] Swain S. S. Sharma D. Hussain T. Pati S. (2020). Molecular mechanisms of underlying genetic factors and associated mutations for drug resistance in *Mycobacterium tuberculosis*. *Emerg. Microb. Infect.* 9 1651–1663. 10.1080/22221751.2020.1785334 32573374 PMC7473167

[B27] Tan T. Lee W. L. Alexander D. C. Grinstein S. Liu J. (2006). The ESAT-6/CFP-10 secretion system of *Mycobacterium marinum* modulates phagosome maturation. *Cell Microbiol.* 8 1417–1429. 10.1111/j.1462-5822.2006.00721.x 16922861

[B28] Tupin A. Gualtieri M. Roquet-Baneres F. Morichaud Z. Brodolin K. Leonetti J. P. (2010). Resistance to rifampicin: at the crossroads between ecological, genomic and medical concerns. *Int. J. Antimicrob. Agents* 35 519–523. 10.1016/j.ijantimicag.2009.12.017 20185278

[B29] Vekemans J. Brennan M. J. Hatherill M. Schrager L. Fritzell B. Rutkowski K. (2020). Preferred product characteristics for therapeutic vaccines to improve tuberculosis treatment outcomes: key considerations from World Health Organization consultations. *Vaccine* 38 135–142. 10.1016/j.vaccine.2019.10.072 31733944

[B30] Vilcheze C. Jacobs W. R. Jr. (2014). Resistance to isoniazid and ethionamide in *Mycobacterium tuberculosis*: genes, mutations, and causalities. *Microbiol. Spectr.* 2:MGM2-0014-2013. 10.1128/microbiolspec.MGM2-0014-2013 26104204 PMC6636829

[B31] World Health Organization [WHO] (2019). *Global Tuberculosis Report 2019.* Geneva: World Health Organization.

[B32] Yong Y. K. Tan H. Y. Saeidi A. Wong W. F. Vignesh R. Velu V. (2019). Immune biomarkers for diagnosis and treatment monitoring of tuberculosis: current developments and future prospects. *Front. Microbiol.* 10:2789. 10.3389/fmicb.2018.02789 31921004 PMC6930807

[B33] Zaw M. T. Emran N. A. Lin Z. (2018). Mutations inside rifampicin-resistance determining region of rpoB gene associated with rifampicin-resistance in *Mycobacterium tuberculosis*. *J. Infect. Public Health* 11 605–610. 10.1016/j.jiph.2018.04.005 29706316

