# Supplementary material for: A Glutamine Insertion at Codon 432 of RpoB Confers Rifampicin Resistance in Mycobacterium tuberculosis
Source: Front Microbiol. 2020 Oct 19;11:583194. doi: 10.3389/fmicb.2020.583194 (PMC7604305; doi:10.3389/fmicb.2020.583194)
Supplement: Supplementary file 1 [file Data_Sheet_1.PDF]

A glutamine insertion at codon 432 of RpoB confers rifampicin resistance in *Mycobacterium tuberculosis*

Li-Yin Lai[1], Li-Yu Hsu[1], Shang-Hui Weng[1], Shuo-En Chung[1], Hui-En Ke[1], Tzu-Lung Lin[1], Pei-Fang Hsieh[1], Wei-Ting Lee[2,3], Hsing-Yuan Tsai[2,3], Wan-Hsuan Lin[2,3], Ruwen Jou[2,3,] and Jin-Town Wang*[1,4]

[1]Department of Microbiology, National Taiwan University College of Medicine, Taipei, Taiwan
[2]Tuberculosis Research Center, Centers for Disease Control, Ministry of Health and Welfare of Taiwan
[3]Center for Diagnostics and Vaccine Development, Centers for Disease Control, Ministry of Health and Welfare of Taiwan
[4]Department of Internal Medicine, National Taiwan University Hospital, Taipei, Taiwan

* Correspondence:
Jin-Town Wang, M.D., Ph.D.
Dept. of Microbiology
National Taiwan University College of Medicine
1, Sec 1, Jen-Ai Rd.
Taipei, Taiwan
wangjt@ntu.edu.tw

Table S1. Primer list

Primer	Sequence (5′-3′)	Purpose
rpoB 432 ins Q-F	CAGCTGAGCCAACAATTCATG	Site-direct mutagenesis
rpoB 432 ins Q-R	CATGAATTGTTGGCTCAGCTG	Site-direct mutagenesis
rpoB 431S 432Q-F	CGCTATAAGGTCAACAAGAAGC	Site-direct mutagenesis
rpoB S431G Q432Q-F	CAGCCAGCTGGGCCAATTCA	Site-direct mutagenesis
rpoB S431G Q432Q-R	TGAATTGGCCCAGCTGGCTG	Site-direct mutagenesis
rpoB G158R-F	CCGAGAAGCGCACGTTCATC	Site-direct mutagenesis
rpoB G158R-R	GATGAACGTGCGCTTCTCGG	Site-direct mutagenesis
rpoB V168A-F	CGAGCGTGCGGTGGTCA	Site-direct mutagenesis
rpoB V168A-R	TGACCACCGCACGCTCG	Site-direct mutagenesis
rpoB S188P-F	GACAAGCCCACCGACAAGAC	Site-direct mutagenesis
rpoB S188P-R	GTCTTGTCGGTGGGCTTGTC	Site-direct mutagenesis
rpoB out F1	CATTCTCACCTGAGGCAACG	PCR and sequencing
rpoB-out-R	ATGATGCCCTTGAAGCGCAC	PCR and sequencing
rpoB out R2	GAAGTACCAGATGTGGGTGAC	PCR and sequencing
rpoB-start-F	TTGGCAGATTCCCGCCAGA	PCR and sequencing
rpoB start-F2	GTGCTGGAAGGATGCATCTTG	PCR and sequencing
rpoB end R	TTACGCAAGATCCTCGACACTT	PCR and sequencing
rpoB 431S 432Q-F	CGCTATAAGGTCAACAAGAAGC	PCR and sequencing
rpoB 431S 432Q-R	TGTAGTCCACCTCAGACGAG	PCR and sequencing
rpoB-F1	TGACCCTCGTTTCGACGATG	PCR and sequencing
rpoB-F2	CTCGTCTGAGGTGGACTACA	PCR and sequencing
rpoB-F3	TCGCTGAAGGTGCCGCAC	PCR and sequencing
rpoB-F4	ACTTGCGCCAACCAGTGCC	PCR and sequencing
rpoB-R	TCGGAGATGTTCGGGATGTC	PCR and sequencing
rpoB-R2	ACATGTAGCCAACCGTGACC	PCR and sequencing
rpoB-R3	TCGGAGATGTTCGGGATGTC	PCR and sequencing
rpoB-R4	CCCTCAGGGGTTTCGATCG	PCR and sequencing
rpoB 170V 188S -F	TAGTCCTAGTCCGAGTCGC	PCR and sequencing
rpoB 170V 188S -R	GCTTCTTGTTGACCTTATAGCG	PCR and sequencing
rpoB 170V-F	AGTCAACGCGAGGACTTGAC	PCR and sequencing
rpoB 170V-R	TGGTCTCGTCGAAGTACACC	PCR and sequencing
